# Higher BMI predicts liver fibrosis among obese children and adolescents with NAFLD - an interventional pilot study

**DOI:** 10.1186/s12887-021-02839-1

**Published:** 2021-09-03

**Authors:** Hadar Moran-Lev, Shlomi Cohen, Muriel Webb, Anat Yerushalmy-Feler, Achiya Amir, Dana L. Gal, Ronit Lubetzky

**Affiliations:** 1 Department of Pediatrics, Dana Dwek Children`s Hospital, affiliated to the Sackler Faculty of Medicine, Tel Aviv University, Tel Aviv, Israel; 2 Pediatric Gastroenterology Unit, Dana Dwek Children’s Hospital, affiliated to the Sackler Faculty of Medicine, Tel Aviv University, Tel Aviv, Israel; 3 Department of Gastroenterology, Tel Aviv Medical Center, affiliated to the Sackler Faculty of Medicine, Tel Aviv University, Tel Aviv, Israel

**Keywords:** Liver, Fibrosis, Steatosis, Obesity, Adolescent

## Abstract

**Background:**

Non-alcoholic fatty liver disease (NAFLD) can range from simple steatosis to steatohepatitis with or without fibrosis. The predictors for liver fibrosis and the effect of nutritional intervention on hepatic fibrosis in pediatric population are not well established. We aimed to investigate the predictors for liver fibrosis and the effects of short-term nutritional intervention on steatosis and fibrosis among obese adolescents with NAFLD.

**Methods:**

Cross-sectional study among obese adolescents. Sociodemographic and clinical data were collected. Liver fibrosis was estimated by Shearwave elastography. All participants were recommended to consume a low carbohydrate diet and were followed biweekly. Blood tests and elastography were performed upon admission and repeated after 3 months.

**Results:**

Fifty-seven pediatric patients were recruited (35 males, mean age 13.5±2.9 years, mean body mass index [BMI] 38.8±9.7). Liver fibrosis was diagnosed in 34 (60%) subjects, which was moderate/severe (F≥2) in 24 (70%). A higher BMI Z score and moderate/severe steatosis correlated with moderate/severe fibrosis (*P* < 0.05). Seventeen patients completed 3 months of follow-up and displayed a decrease in BMI Z score (from BMI Z score 2.6±0.5 before intervention to 2.4±0.5 after intervention), with a significant decrease in liver fibrosis (*P* = 0.001).

**Conclusion:**

Pediatric patients with high BMIs and severe liver steatosis are at risk for severe liver fibrosis. Nutritional intervention with minimal weight loss may improves hepatic fibrosis among the pediatric population.

**Trial registration:**

TRN NCT04561804 (9/17/2020)

## Background

Non-alcoholic fatty liver disease (NAFLD) is the most common etiology of chronic liver disease in adults and children in the developed world [1], with a prevalence of 3-10% in the general pediatric population and up to >70% in obese children [2, 3]. NAFLD encompasses a wide spectrum of histological and clinical manifestations, ranging from simple steatosis with debatable clinical significance to non-alcoholic steatohepatitis (NASH), with or without fibrosis that may develop into cirrhosis and liver failure, even in young children [3]. Therefore, in view of the increased prevalence of obesity among children and adolescents, it is of upmost importance to identify young patients at risk for advanced fibrosis who may develop cirrhosis and liver failure. Only few studies have aimed to find predictors for advanced fibrosis in pediatric NAFLD patients [4–6]. Moreover, the current mainstay of treatment for both adult and pediatric NAFLD is weight loss, but the effect of dietary intervention on hepatic fibrosis in pediatric population is not well established [7, 8].

The aims of the present study were to investigate predictors for liver fibrosis in obese pediatric patients, and to assess the effects of short-term dietary intervention on steatosis and fibrosis.

## Methods

### Patient population

We prospectively recruited all children and adolescents (age 7-18 years) with obesity who were admitted to the Obesity Clinic at Dana-Dwek Children’s Hospital of the Tel Aviv Medical Center between December 1, 2018, and December 1, 2019. All children with a BMI >95 percentile for age were included in the study. Patients with a diagnosed primary liver disease (e.g., autoimmune liver disease, metabolic liver disease, Wilson`s disease, alpha 1 antitrypsin deficiency), patients treated with medications known to induce steatosis (e.g., valproate, amiodarone or prednisone), and patients with hepatic virus infections or history of parenteral nutrition were excluded from the study.

### Study design and measurements

This study is part of a clinical trial that assessed the effect of bariatric surgery and dietary intervention on hepatic fibrosis in an obese pediatric population with NAFLD (Clinical Trial Registration NCT04561804). At the initial visit, data were collected on socioeconomic parameters, lifestyle, birth details, and medical, family, and social histories. All patients underwent anthropometric measures (height, weight, and BMI) and a physical examination focused on obesity-related conditions. Laboratory evaluation included liver enzyme profile, lipid profile, and glucose, insulin, and HbA1C levels. Liver fibrosis was estimated by Shearwave elastography (Supersonic) and categorized into 4 levels, F0-F4, according to liver stiffness (measured by kPa), as recently demonstrated elsewhere [9]. Liver steatosis was calculated by a hepatorenal index (HRI), as described by Webb et al [10], and divided into 3 levels of severity. All measurements were taken by a single experienced radiologist (MW), who was blinded to the results of other parameters of the patients. A multidisciplinary team included a gastroenterologist, hepatologists, a registered dietitian (RD) and a psychologist. All participants received nutritional recommendations (see below) and general recommendations for a healthy lifestyle (regular engagement in daily physical activity and reduction of screen time). Compliance with the dietary guideline was reviewed by an RD on a biweekly basis with a 3-day food questionnaire (2 weekdays and 1 day of the weekend). Blood tests and elastography were repeated after 3 months of intervention.

Ethnicity was defined as the birthplace of the parents or grandparents (if the parents were born in Israel) and categorized according to country of origin. Determination of the BMI percentiles for age and sex was based on WHO growth charts. "Obesity” was defined as a BMI >95th percentile [11]. Abnormal glucose metabolism included taking a hypoglycemic medication or having an elevated homeostatic model assessment index of insulin resistance, glucose, or HBA1c. Hypertriglyceridemia and hypercholesterolemia were defined as a serum level >95 percentile for age and sex [12]. Hypertension was diagnosed as systolic and/or diastolic blood pressure ≥95 percentile for age and sex [13]. Patients with clinical suspicion of obstructive sleep apnea (OSA) were diagnosed by polysomnography that was conducted by the hospital`s sleep specialists. Socioeconomic status was defined according to parents` years of education.

### The dietary intervention

The participants received nutritional recommendations for a low carbohydrate, low glycemic load, and isocaloric diet. The diet was composed of carbohydrates (CHO;30-40%), fats (35-50%), and proteins (20-25%), and was tailored to individual preferences and calorie requirements. The number of CHO, protein, and fat servings was determined according to the recommended total energy requirements for age, calculated on the basis of dietary reference intake (DRI). Participants were not instructed to restrict calories, but to reduce carbohydrates based on their glycemic load. High glycemic index (GI) carbohydrate intake (refined grains, potatoes, sweet and salted snacks, and sugar sweetened beverages) was completely restricted, low GI carbohydrates (non-starchy vegetables, legumes, nuts,) were allowed, and some low/moderate GI carbohydrates such as fruits and whole grain bread were allowed but limited. The subjects were instructed about appropriate food choices, and each participant was provided a diet information booklet containing a food list, sample menus, and recipes.

### Statistical analyses

Descriptive statistics were examined for all variables. Continuous variables were expressed as median with interquartile range (IQR) when they were not normally distributed and as mean ± standard deviation (SD) for normally distributed variables. Categorical variables were presented as number and percentage. Categorical variables were compared by the chi-square test or Fisher’s exact test, and continuous and ordinal variables by the Wilcoxon test. The Fisher test was used when the McNamer test was not applicable for some variables. The Pearson correlation and simple linear regression analysis were performed to examine bivariate associations between fibrosis and metabolic and nutritional parameters. The Wilcoxon signed rank test was applied to compare the difference between steatosis, fibrosis, and metabolic parameters between the 2 time points (baseline and 3-month follow-up). A *P* level <0.05 was considered statistically significant. All statistical tests were 2-sided. The statistical analysis was performed with SPSS (IBM SPSS statistics, version 22, IBM Corp. Armonk, NY, USA, 2013.).

### Ethical considerations

The study protocol was approved by the institutional review board of the medical center (TLV-0097-17). Signed informed consent was obtained from the parents of all the participants. The study was design in accordance to the CONSORT guidelines.

## Results

### Description of overall study sample

**Fig. 1:**
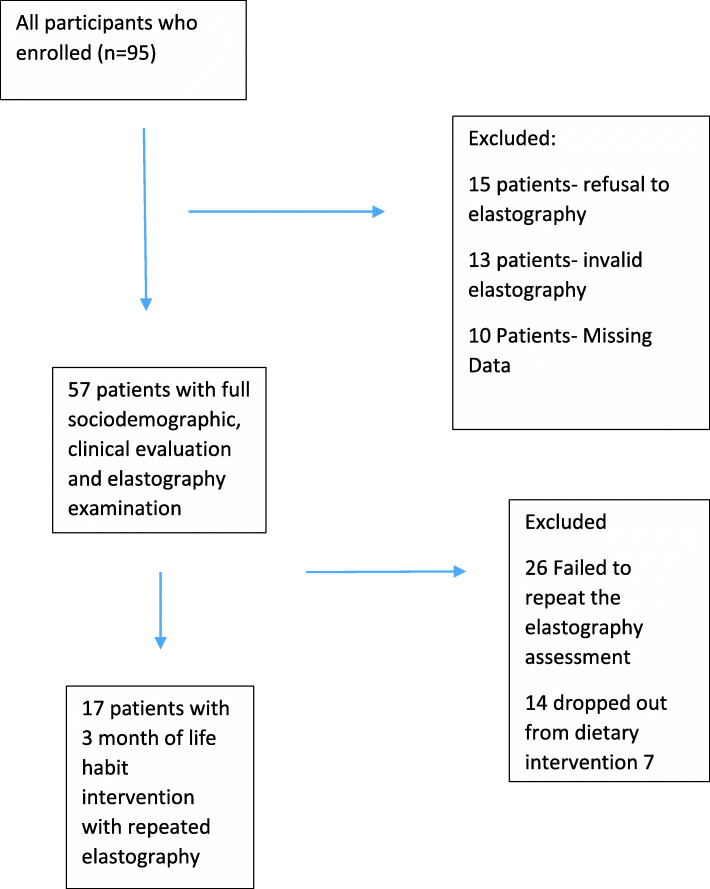
Determination of study sample

Ninety-five consecutive children and adolescents with obesity were recruited. Fifteen patients were excluded for refusal to undergo elastography examination, 13 patients were excluded for invalid elastography examinations, and 10 patients were subsequently excluded due to missing data. Figure 1 provides a flowchart depicting patient selection. The final cohort consisted of 57 patients [35 (61%) males, with a mean age of 13.5±2.9 years and a mean BMI of 38.8±9.7 (Table 1). Their ethnic distribution revealed that 47% of the patients were Jews of mixed origin (Ashkenazi and Sephardi), 23% were Sephardi Jews, 21.9% were Ashkenazi Jews, and 8.1% were Arabs. Baseline blood tests demonstrated impaired fasting glucose in 22 subjects (39%), elevated triglycerides in 26 (45%), and hypercholesterolemia in 14 (25%). Hypertension and OSA were documented in 5 patients (9% each) (Table 1). Fifty-three (92%) subjects were diagnosed with liver steatosis upon admission to the clinic. A total of 34 (60%) patients had liver fibrosis which was moderate/severe (F≥2) in 24 (70%) of them

**Table 1:** Demographic and clinical characteristics of the study population

Characteristics	Mean±SD
Age, years	13.5±2.9
Sex, male, n (%)	35 (61.4)
BMI (kg/m^2^)	38.8±9.7
BMI Z score	2.6±0.5
HbA1c	5.3±0
Glucose (mg/dL)	91.6±9.9
HOMA-IR	9.8±7.1
ALT (U/L)	46±37.9
LDL (mg/dL)	106.2±36.2
Hypercholesterolemia, n (%)	14 (25%)
HDL (mg/dL)	41.4±11.1
Triglyceride (mg/dL)	124.6±56.8
Hypertriglyceridemia, n (%)	26 (45%)
Hypertension, n (%)	5 (9%)
Obstructive sleep apnea, n (%)	5 (9%)

*SD* standard deviation; *BMI* body mass index; *ALT* alanine transaminase; *LDL* low-density lipoprotein, *HDL* high-density lipoprotein; *HOMA* Homeostatic model assessment for insulin resistance

### Predictors for liver steatosis/fibrosis

A comparison between subjects with moderate/severe fibrosis (F≥2) and those with minimal or no fibrosis (F<1) revealed some significant group differences. Higher BMI levels were significantly more prevalent in subjects with fibrosis levels of ≥F2 compared to subjects with minimal or no fibrosis (43.8±9.5 vs 34.9±8, respectively, *P* < 0.001) (Table 2). In addition, moderate/severe steatosis was more frequent in subjects with fibrosis levels of ≥F2 compared to subjects with minimal or no fibrosis (67% vs. 32%, *P* < 0.001, HRI 2.1±0.4 vs HRI 1.8±0.5, respectively, *P* = 0.02). Among the metabolic parameters, the low-density lipoprotein (LDL) was significantly lower in subjects with moderate/severe fibrosis (92.4±29.3 mg/dL vs 116.7±38.2 mg/dL for patients with F≥2 vs F≤1, respectively, *P* = 0.04). There were no significant differences in socioeconomic status, perinatal factors (mode of delivery, birth weight, breastfeeding), age of adiposity rebound, sex and other metabolic parameters (triglyceride, HDL, and LDL levels, HgBA1C, liver enzyme) between the two groups. There was a trend towards a higher mean age in subjects with significant fibrosis (14.3 vs. 12.9 years, *P* = 0.058). The Pearson correlation revealed a strong association between steatosis and liver fibrosis (r=0.65, *P* = 0.001), BMI, and liver fibrosis (r=0.4, *P* = 0.001). and an inverse association between serum cholesterol levels and liver fibrosis (r= -0.4, *P* = 0.01).

**Table 2:** Comparison between Subjects with Mild or no Fibrosis (F≤1) and Subjects with Moderate/Severe Fibrosis (F≥2)

	Fibrosis ≤1 n = 33	Fibrosis ≥2 n = 24	*P* Value
Age, year	12.9±2.8	14.3±2.8	0.058
Male (%)	22 (66)	13 (54)	NS
Birth weight (kg)	2.9±6.5	2.9±8	NS
Breastfeeding (%)	11 (34)	6 (26)	NS
High SES (%)	13 (41.7)	9 (41.9)	NS
Age at adiposity rebound	6.7±2.8	5.2±2.7	NS
BMI	34.9±8	43.8±9.5	<0.001
BMI Z score	2.4±0.3	2.8±0.6	0.004
Triglycerides, mg/dL	133±61	112.9±49	NS
LDL, mg/dL	116.7±38.2	92.4±29.3	0.04
HDL, mg/dL	40.9±10.3	42.1±12.2	NS
HbA1c	5.3±0.3	5.4±0.5	NS
OSA (%)	2(4)	3(19)	NS
ALT, U/L	39.3±29.8	52.8±44.4	NS
Moderate/severe steatosis, n (%)	10 (32)	21 (67)	<0.01
HRI	1.8±0.5	2.1±0.4	0.02

Values are expressed as mean and standard deviation (SD) or %

*BMI* body mass index; *ALT* alanine transaminase; *LDL* low-density lipoprotein; *HDL* high-density lipoprotein, *HRI* hepatorenal index; *SES* socioeconomic status; *OSA* Obstructive sleep apnea

### The effect of short-term life habit intervention on liver steatosis and fibrosis

**Fig. 2:**
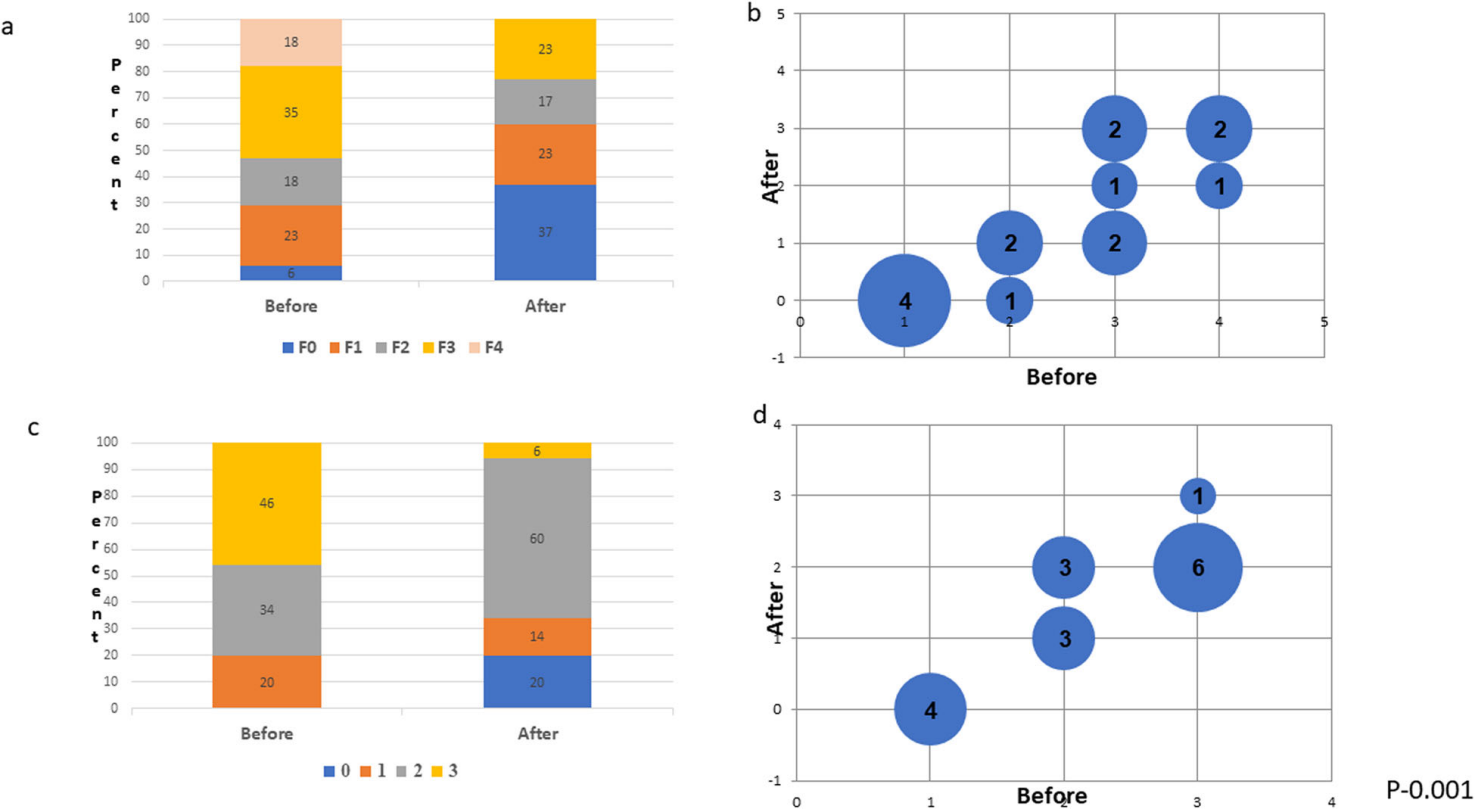
Differences in (**a**) percent of fibrosis distribution, (**b**) fibrosis stage, (**c**) percent of steatosis distribution, and (**d**) steatosis stage before and after dietary intervention

Seventeen patients completed 3 months of follow-up with repeated blood tests and elastography (11 males, 6 females, mean age 13.8±2.5 years, Fig. 1). Table 3 shows the average of the participants’ self-reported dietary intake before the nutritional intervention and at the midpoint of the 3 months of intervention. Before the nutritional intervention, the average caloric consumption for the 7–13-year age group was 1939 kcal/day, of which 54% was derived from carbohydrates, 15% from protein, and 30% from fat. After the dietary intervention, the average caloric consumption was 1984 Kcal/day, consisting of 30%, 25% and 45% energy from CHO, protein, and fat, respectively. For the 14-18 years age group, the average reported caloric consumption before the nutritional intervention was 2374 kcal/day, of which 48% was derived from carbohydrates, 20% from protein, and 32% from fat. After the dietary intervention, the average caloric consumption was 2039 Kcal/day, consisting of 30%, 25%, and 45% energy from CHO, protein, and fat, respectively. Although participants were not instructed to restrict calories, we noticed a significant decrease in the BMI z score (from 2.6±0.5 before intervention to 2.4±0.5 after the intervention (Table 4, *P* = 0.008). There was also a significant decrease in the incidence of liver fibrosis and steatosis after the nutritional intervention (Fig. 2). Before the dietary intervention, 3 patients (18%) had F4 fibrosis, 5 (35%) had F3 fibrosis, 3 (18%) had F2 fibrosis, and 4 (23%) had F1 fibrosis. After the dietary intervention, none of the patients had F4 fibrosis. The level of fibrosis decreased to F2 in 1 patient with F4 and to F2 in 2 patients with F4. The fibrosis decreased from F3 to F2 in 1 patient with ???and from F3 to F1 in 2 patients with F3 fibrosis. Among the 3 patients with F2 fibrosis, the level of fibrosis decreased to F1 in 2 and to F0 in one. There were 4 patients with F1 who had complete normalization of the fibrosis after the dietary intervention. (*P* = 0.001, Fig. 1a-b). A similar improvement was also noted in the liver fat content as measured by the HRI (Fig. 1 d-c). These changes were also accompanied by a significant decrease in ALT and triglyceride serum levels (from 61±34 mg/dl before intervention to 42±26.4 mg/dl after intervention and from 147.6±68 mg/dl before intervention to 102.2±44.4 mg/dl after intervention, respectively), with no significant difference in HDL or LDL levels (from 42.1±16.1 mg/dL to 43±18.1 mg/dL and from 143.2±58.2 mg/dL to 102.7±65 mg/dL, respectively) (Table 4).

**Table 3:** Self-reported dietary intake before and after the nutritional intervention

Age	Variable	Amount	Before Intervention n = 17	After Intervention n = 17
7-13 years	Total energy	kcal	1939±390	1984±423
	Carbohydrates	% Kcal	55±4.4	30±5.9
		g/day	262±65	150±78
	Protein	% Kcal	15±2.9	25±6.4
		g/day	72±20	125±23
	Fat	% Kcal	31±4.2	45±4.4
		g/day	63±18	99±15
	Saturated Fat	% Kcal	12±1.9	13±2.2
		g/day	26±6	28±4
	Sodium	(mg)	3756±675	2187±32
14-18 years	Total energy	kcal	2374±410	2039±450
	Carbohydrates	% Kcal	48±6.6	35±7
		g/day	274±350	178±34
	Protein	% Kcal	20±4.8	21±6.3
		g/day	120±32	106±32
	Fat	% Kcal	32±3.3	46±8.2
		g/day	85±22	105±45
	Saturated Fat	% Kcal	8.7±2.3	11.4±3.2
		g/day	23±2.5	26±2.1
	Sodium	mg	4252±453	4100

Data reported as Mean±SD

Reports are average reported intakes from 3-day food records

**Table 4:** Differences in metabolic parameters after dietary intervention

Variable	Before Intervention n = 17	After Intervention n = 17	*P* Value
BMI Z Score	2.6±0.5	2.4±0.5	0.008
Weight (kg)	114.3±33.7	106.9±33.8	0.06
ALT (mg/dL)	61±34	42±26.4	0.002
LDL (mg/dL)	143.2±58.2	102.7±65	NS
Triglyceride (mg/dL)	147.6±68	102.2±44.4	0.001
HDL (mg/dL)	42.1±16.1	43±18.1	NS
HbA1c	5.3±0.4	5.3±0.3	NS
Glucose (mg/dL)	93.5±9.4	92.8±9.4	NS
Liver fibrosis - kPa	8.9±2.4	7.4±1.4	0.006

*BMI* body mass index; *ALT* alanine transaminase; *LDL* low-density lipoprotein; *HDL* high-density lipoprotein; *kPa* kilopascals

Values are given ± standard deviation

## Discussion

The results of the present study indicate that higher BMI levels and moderate/severe liver steatosis are predictors for severe liver fibrosis in children and adolescents. Three months of nutritional intervention based on a low carbohydrate diet improved hepatic steatosis and fibrosis in a pediatric population with NAFLD.

Our data corroborate with those of others [3, 7, 14, 15] by demonstrating a high rate of NAFLD with a significant percent of moderate to severe fibrosis in morbidly obese young individuals, reaching approximately 70% of our patients. Only few attempts have been made to stratify the risk for advanced fibrosis in this unique population [6–8]. Moreover, recent data have suggested that adult scores may not be accurate to predict advanced fibrosis in children [7, 16, 17], thus establishing a clear need to evaluate noninvasive approaches in children as well. The pediatric NAFLD fibrosis index is based on age, waist circumference, and triglycerides, and it has been described by Nobili et al as a possible tool to predict liver fibrosis in children [4]. It is, however, limited by not including children with moderate/severe fibrosis. The recent pediatric NAFLD fibrosis score which included ALT, alkaline phosphatase, platelet counts, and gamma glutamyl transferase levels was reported to predict the presence of significant fibrosis, but it lacks external validation [6].

Our current results demonstrated that moderate/severe fibrosis correlated with higher BMI levels and moderate/severe steatosis. This reinforces previous findings which demonstrated that children and adolescent with severe obesity (BMI ≥120% of the 95^th^ percentile or an absolute BMI ≥35 kg/m^2^) are more prone to severe complications, such as cardiovascular disease, dyslipidemia and inflammation [18, 19] compared to children and adolescents with obesity and lower BMI levels. This highlights the need for early dietary intervention, even among youngsters, before further complications develop and the severity increases.

The only metabolic parameter that was significantly related to moderate/severe fibrosis was lower LDL. Moreover, we found a trend for higher triglyceride levels among patients with lower fibrosis levels. These results may reflect the recent NASH Clinical Research Network data which demonstrated that zone 1 steatosis, while rare in adult populations, was highly prevalent in children with NAFLD, and that it represents a distinct sub-phenotype with unique metabolic and histologic parameters. Children with zone 1 steatosis had lower fasting triglyceride levels and lower fasting insulin according to the NASH report. However, zone 1 steatosis was found to have more fibrosis of any grade (81% vs 51) and more advanced fibrosis (13% vs 5%) compared to children with zone 3 steatosis [20]. Our findings did not include biopsy data, but these unique differences in metabolic parameters between subjects with moderate/severe fibrosis to patients with minimal or no fibrosis may also serve to emphasize the need for early intervention in NAFLD patients even if no other metabolic disorder is present.

Seventeen of our patients completed 3 months of follow-up with dietary interventions, repeated blood tests, and elastography. There was a significant decrease in the BMI Z score, with a significant decrease in liver fibrosis and steatosis scores at the end of follow-up. Moreover, ALT and triglyceride serum levels decreased significantly as well. There are several possible explanations for the significant restitution of liver fibrosis that was demonstrated in our study after only 3 months. First, it may be due to the weight loss itself that was documented in our cohort. Reduction of visceral fat depots after weight loss protects against the overflow of fatty acids to the liver [21, 22]. Increased availability of fatty acid, in turn, is pivotal to the pathogenesis of fatty liver, causing mitochondrial dysfunction and lipotoxicity [22]. Second, it may be due to the specific dietary intervention. The change in liver fat in our study occurred without major weight loss. This was also described in other studies of adults and children [21–23], suggesting the possibility of clinical benefit solely with low carbohydrate dietary modification, since a lower glycemic response causes less hepatic glucose absorption [24–26]. Several clinical trials demonstrated that a reduction of fructose or sugar consumption resulted in lower intrahepatic fat, lipogenesis, inflammation, and insulin resistance [24–26]. Moreover, because this diet does not restrict either fat or protein, it may also be more behaviorally sustainable and can therefore result in better adherence over time [27]. Lastly, it could be that the rapid and significant reversal in liver histology, compared to the adult population, stems from the differences in histologic distribution among the 2 populations in terms of inflammation and hepatocellular damage [16, 20, 28].

The main limitation of our study is the lack of liver biopsies for assessing NAFLD, which is still considered the gold standard for NAFLD diagnosis. However, the well-known limitations of liver biopsy and the fact that liver biopsy cannot be applied to all patients suspected of having NAFLD have led to the development of noninvasive methods for the assessment of liver fibrosis. Shear-wave elastography was recently shown to be an accurate and reproducible noninvasive technique that efficiently depicts the presence of liver fibrosis in the pediatric population with NAFLD [9, 17], with high levels of repeatability and reproducibility and high intra-observer (ICC = 0.89–0.90) and inter-observer (ICC = 0.81–0.85) coefficients [29, 30]. Other limitations of our study are the lack of a control group and the 3-month follow-up period that may not have been long enough to observe the full extent of influence of macronutrient contents on NAFLD and fibrosis. Nevertheless, the prospective nature of this study and the significant improvement that was demonstrated after 3 months in the repeated elastography taken together with the significant decrease in ALT and triglyceride serum levels enables us to draw important conclusions about the need for early intervention in the obese pediatric population with NAFLD, and be encouraged by the results that testify to the ability of histological improvement if appropriate treatment is offered in time.

## Conclusion

Our study findings reveal that a higher BMI carries a greater risk for advanced liver fibrosis in the pediatric population. A low carbohydrate and low glycemic index diet may improve hepatic steatosis and fibrosis already after a 3-month period. Longitudinal and larger-cohort studies are needed to compare the effectiveness of a low carbohydrate diet with that of other dietary interventions for preventing the progression of NAFLD toward more severe forms of liver derangements early in its natural history.

## Data Availability

The datasets used and/or analyzed during the current study are available from the corresponding author on reasonable request.

## Abbreviations

BMI: Body mass index
ALT: Alanine transaminase
LDL: Low-density lipoprotein
HDL: High-density lipoprotein
HRI: Hepatorenal index
SES: Socioeconomic status
kPa: kilopascals
CHO: Carbohydrates
NAFLD: Non-alcoholic fatty liver disease
NASH: Non-alcoholic steatohepatitis
CHO: Carbohydrates
RD: Registered dietitian
OSA: Obstructive sleep apnea
GI: Glycemic index
